# Supplementary material for: PRP4 Induces Epithelial–Mesenchymal Transition and Drug Resistance in Colon Cancer Cells via Activation of p53
Source: Int J Mol Sci. 2022 Mar 13;23(6):3092. doi: 10.3390/ijms23063092 (PMC8955441; doi:10.3390/ijms23063092)
Supplement: Supplementary file 1 [file ijms-23-03092-s001.zip › Supplementary Table S1.pdf]

Table S1. List of all PRP4 regulated miRNAs. 84 miRNAs were found to be regulated by PRP4 over-expression in HCT116 cells.

RQ	CTRL	PRP4	RQ	CTRL	PRP4	RQ	CTRL	PRP4
mir-151a-5p	1	1.378405	mir-142-5p	1	1.850609	mir-194-5p	1	1.623379
mir-195-5p	1	1.233707	mir-9-5p	1	1.199971	mir-210	1	1.79005
mir-143-3p	1	1.585568	mir-150-5p	1	1.276329	mir-15a-5p	1	1.276329
mir-30d-5p	1	1.367935	mir-27b-3p	1	1.408344	mir-181a-5p	1	1.470187
mir-191-5p	1	1.251796	mir-101-3p	1	1.44093	mir-15b-5p	1	1.223488
mir-let.7i.5p	1	1.18756	let-7d-5p	1	1.360371	mir-99a-5p	1	1.155887
mir-302a-3p	1	1.209156	mir-103a-3p	1	1.220947	mir-28-5p	1	1.351911
mir-222-3p	1	1.188383	mir-16-5p	1	1.188383	mir-320a	1	1.351911
let-7b-5p	1	1.415194	mir-26a-5p	1	1.272795	mir-125-5p	1	1.366987
mir-19b-3p	1	1.147902	mir-32-5p	1	2.020903	mir-29b-3p	1	1.489677
mir-17-5p	1	1.007654	mir-26b-5p	1	1.181812	mir-29a-3p	1	1.232852
mir-93-5p	1	1.111109	let-7g-5p	1	1.22264	mir-141-3p	1	1.549715
mir-186-5p	1	1.089752	mir-30c-5p	1	1.255272	mir-19a-3p	1	1.166349
mir-196b-5p	1	1.190032	mir-96-5p	1	1.693491	mir-18a-5p	1	0.925304
mir-27a-3p	1	1.336074	mir-185-5p	1	1.350038	mir-374a-5p	1	1.088997
mir-22-3p	1	1.223488	mir-142-3p	1	2.242332	mir-423-5p	1	1.293249
mir-130a-3p	1	1.344435	mir-24-3p	1	1.309485	let-7a-5p	1	1.356604
let-7c	1	1.432962	mir-155-5p	1	2.005553	mir-124-3p	1	1.491744
mir-29c-3p	1	1.293249	mir-146a-5p	1	2.108183	mir-92a-3p	1	1.177723
mir-140-3p	1	1.311302	mir-425-5p	1	1.095812	mir-23a-3p	1	1.416175
mir-128	1	1.151888	mir-181b-5p	1	1.297739	mir-25-3p	1	1.083726
let-7f-5p	1	1.309485	mir-302b-3p	1	1.370783	let-7e-5p	1	1.260503
mir-122-5p	1	1.125058	mir-30b-5p	1	1.353786	mir-376c-3p	1	1.614402
mir-20a-5p	1	1.018891	mir-21-5p	1	1.524145	mir-126-3p	1	1.338855
mir-106b-5b	1	1.055554	mir-30e-5p	1	1.345367	mir-144-3p	1	0.893785
mir-7-5p	1	1.054091	mir-200c-3p	1	1.081475	mir-424-5p	1	1.417157
mir-100-5p	1	1.128964	mir-15b-5p	1	1.164734	mir-30a-5p	1	1.343503
mir-302c-3p	1	1.253533	mir-223-3p	1	1.796265	mir-23b-5p	1	1.349103
