# Supplementary material for: PRP4 Induces Epithelial–Mesenchymal Transition and Drug Resistance in Colon Cancer Cells via Activation of p53
Source: Int J Mol Sci. 2022 Mar 13;23(6):3092. doi: 10.3390/ijms23063092 (PMC8955441; doi:10.3390/ijms23063092)
Supplement: Supplementary file 1 [file ijms-23-03092-s001.zip › Supplementary Table S2.pdf]

Table S2. List of PRP4 upregulated miRNAs. 13 miRNAs were upregulated by PRP4 transfection to HCT116 cells (RQ value > 1.5). The threshold we used to screen up- or downregulated miRNAs was a fold-change ≥ 1.5 or ≤ 1.0.

RQ	CTRL	PRP4
mir-142-3p	1	2.24
mir-146a-5p	1	2.108
mir-32-5p	1	2.021
mir-155-5p	1	2.006
mir-142-5p	1	1.851
mir-223-3p	1	1.796
mir-210	1	1.790
mir-96-5p	1	1.693
mir-194-5p	1	1.623
mir-376c-3p	1	1.614
mir-143-3p	1	1.586
mir-141-3p	1	1.550
mir-21-5p	1	1.524
