# Supplementary material for: PRP4 Induces Epithelial–Mesenchymal Transition and Drug Resistance in Colon Cancer Cells via Activation of p53
Source: Int J Mol Sci. 2022 Mar 13;23(6):3092. doi: 10.3390/ijms23063092 (PMC8955441; doi:10.3390/ijms23063092)
Supplement: Supplementary file 1 [file ijms-23-03092-s001.zip › Supplementary Table S3.pdf]

Table S3. List of PRP4 downregulated miRNAs. 2 miRNAS were downregulated by PRP4 over-expression in HCT116 cells. RQ value < 1.

RQ	CTRL	PRP4
mir-18a-5p	1	0.925304
mir-144-3p	1	0.893785
