# Supplementary figures and images for: PRP4 Induces Epithelial–Mesenchymal Transition and Drug Resistance in Colon Cancer Cells via Activation of p53
Source: Int J Mol Sci. 2022 Mar 13;23(6):3092. doi: 10.3390/ijms23063092 (PMC8955441; doi:10.3390/ijms23063092)

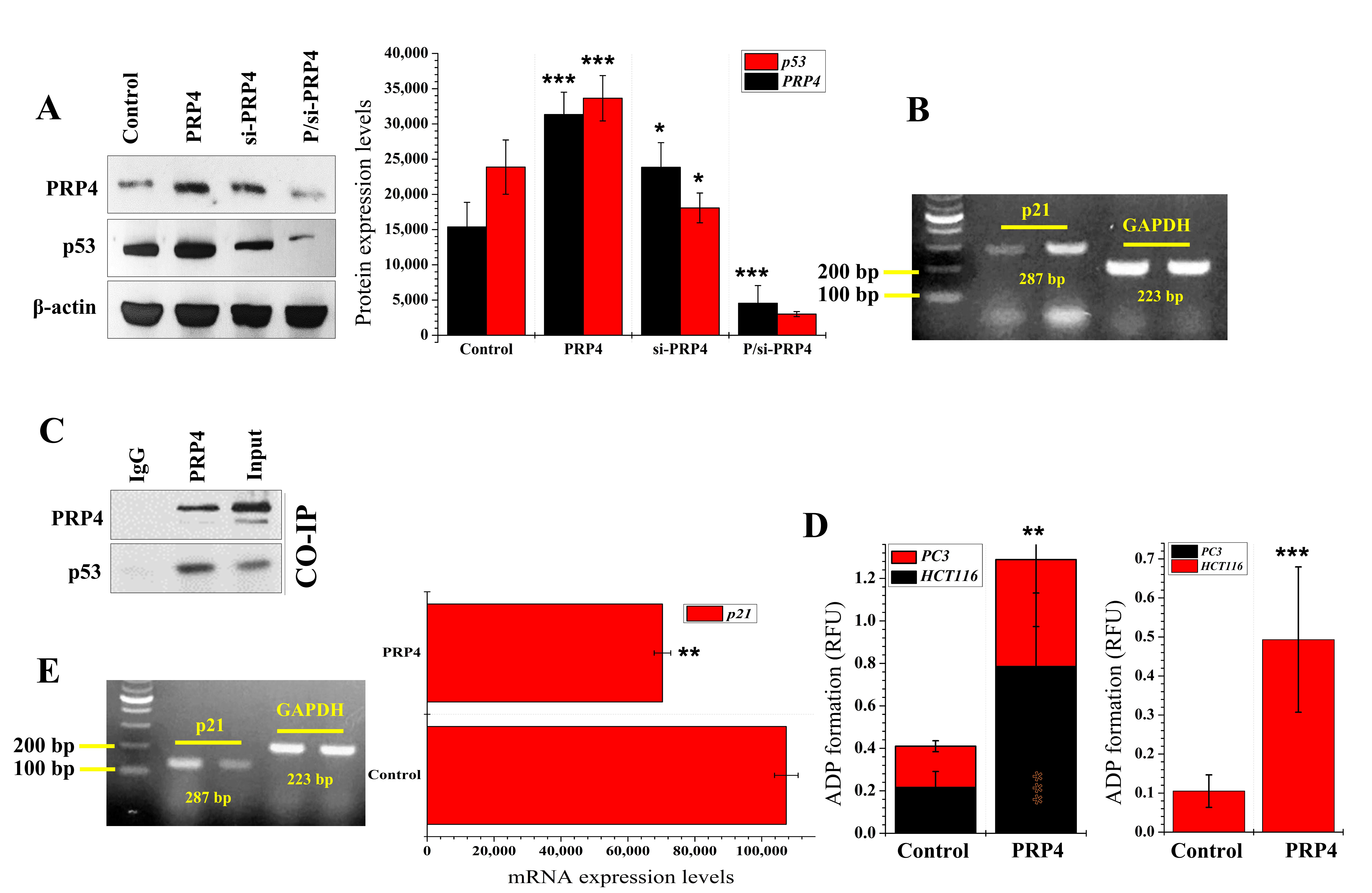

Supplement: Supplementary file 1 [file ijms-23-03092-s001.zip › Supplemental Figure S1.jpg]

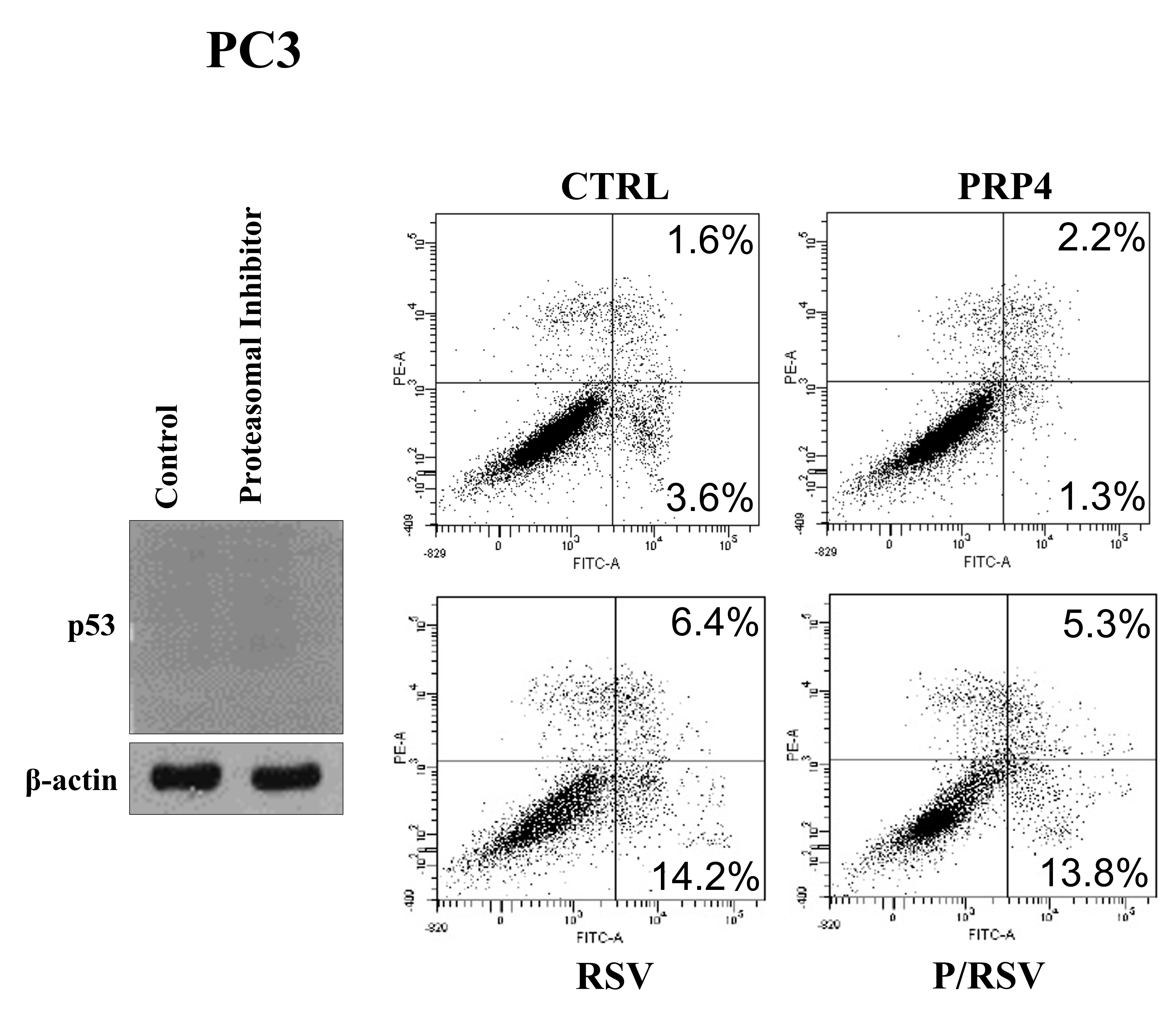

Supplement: Supplementary file 1 [file ijms-23-03092-s001.zip › Supplemental Figure S2.jpg]
